# Attitudes towards primary care career in community health centers among medical students in China

**DOI:** 10.1186/s12875-016-0472-5

**Published:** 2016-07-16

**Authors:** Lingling Zhang, Thomas Bossert, Ajay Mahal, Guoqing Hu, Qing Guo, Yuanli Liu

**Affiliations:** Department of Public Health Sciences, Clemson University, 515 Edwards Hall, Clemson, SC 29634 USA; Department of Global Health and Population, Harvard T.H. Chan School of Public Health, 665 Huntington Avenue, Boston, MA 02115 USA; Monash University School of Public Health and Preventive Medicine, Welling Road, Clayton, Victoria 3800 Australia; Xiangya School of Public Health, Central South University, 110 Xiangya Road, Changsha, 410078 China; Zhejiang Chinese Medical University; Intelligent Health Management Institute, Hangzhou Normal University, 16 Xuelin St, Jianggan, Hangzhou 310036 China; Peking Union Medical College School of Public Health, Beijing, 100730 China

**Keywords:** Career choice, Primary health care, General Physicians, Medical student, Community health center, China

## Abstract

**Background:**

Very few of the primary care doctors currently working in China’s community health centers have a college degree (issued by 5-year medical schools). How to attract college graduates to community services in the future, therefore, has major policy relevance in the government’s ongoing efforts to reform community health care and fill in the long-absent role of general physicians in China. This paper examined medical school students’ attitudes towards working in communities and the factors that may affect their career choices in primary care to inform policy on this subject.

**Methods:**

A cross-sectional survey was designed upon the issuance of community health reform policy in 2006 by the Chinese government*.* The survey was conducted among 2714 medical students from three medical schools in representative regions in China. Binomial and multinomial regression analyses were carried out using a collection of plausible predictors such as place of rearing, income, etc. to assess their willingness to work in communities.

**Results:**

Of the 2402 valid responses, besides 5.7 % objection to working in communities, 19.1 % expressed definite willingness. However, the majority (41.5 %) of students only consider community job as a temporary transition, in addition to 33.7 % using it as their backup option. The survey analyses found that medical students who are more likely to be willing to work in communities tend to come from rural backgrounds, have more exposure to community health reform, and possess certain personally held value and fit.

**Conclusion:**

To attract more graduates from 5-year medical schools to work in communities, a targeted recruiting approach or admission policy stands a better chance of success. The findings on the influencing factors of medical students’ career choice can help inform policymakers, medical educators, and community health managers to improve the willingness of swing students to enter primary care to strengthen basic health services.

**Electronic supplementary material:**

The online version of this article (doi:10.1186/s12875-016-0472-5) contains supplementary material, which is available to authorized users.

## Background

Effective primary care have been shown to be associated with improved access to health care services and population health, cost effectiveness, and enhanced equity [1–4]. A key element of China’s recent health system reform efforts is building of a primary care safety net at the level of the local community with the hope that community doctors in China will eventually become the “gate-keepers” of the health of the population with a role similar to the General Physicians (GPs) in more developed countries. Community health centers (CHC), thus has become the foundation of providing primary health care services in China. In 2006, the State Council promulgated “*Guidelines on the Development of Urban Community Health Services*” [No.10 (2006) of the State Council] and “*Guidelines on Strengthening the Role of Urban Community Health Personnel*” [No. 69 (2006) of the Ministry of Human Resources and Social Security]. In 2011, the State Council further announced the goal of achieving 2–3 qualified GPs for every 10,000 residents in both urban and rural China by 2020. [No. 23 (2011) of the State Council] But less than 13 % of health care providers held a bachelor’s degree in medicine or higher in community health centers in China [5]. The government has attempted to upgrade these health care providers to be a fully GP with a bachelor degree and promote community health services. However, China is facing a paradox where urban oversupply and rural scarcity in health professionals coexist [6], which has been seen in developed countries as well [7, 8]. Despite the governments’ efforts at tackling this issues, many medical graduates with a bachelor’s degree in medicine or higher still chose a career of becoming a specialist [8], which leads a severe shortage of health providers with desired academic training in both urban and rural community health services.

Unlike countries such as the US, Canada, and Australia where medical education is a graduate-level program, medical education in China is an undergraduate-entry program with a variety of levels including medical schools that offer 3, 5, 7, or 8 years of education after high school. Medical students in China may obtain their medical degrees through different educational programs (a 3-year training program for a diploma certificate, 5-year program for a bachelor’s degree, 7-year program for a master’s degree and an 8-year doctor degree program) [9]. As a bachelor degree is the desired educational level for GPs’ qualification in China, in this study, we surveyed 2402 medical students enrolled in 5-year training programs to assess their willingness to work in communities and the factors likely to influence that willingness. The existing literature on medical students’ career choice found that the influencing factors include gender [10, 11], place of rearing [12, 13] and prior contact [10, 14–16]. However, very limited data were available in public literature regarding potential factors that may influence career choice in primary care among medical students in China.

The objective of this study is to examine the factors that affect medical students’ career choice in general medicine and contribute to the current knowledge of the attitudes of medical students towards working in primary care settings.

## Methods

### Study population

Upon the issuance of *Guidelines* [No.10 (2006) of the State Council], we used a stratified sampling method to select three medical schools from three provinces in eastern region (School I), central region (School II), and western region (School III) of China according to the conventional classification of economic development ranging from high to low, respectively. All these three medical schools provide 5-year medical training. In each school, medical students in all five grades were surveyed except for fourth-year students in School I, who were unavailable owing to duties linked to clinical rotations outside the school. Paper-based questionnaires were distributed to students during a class gathering and they filled out the questionnaire anonymously. This survey was conducted following the principles of research ethics. Approval of Institute Review Board was exempt given the nature of this study (an anonymous survey that no survey participant can be identified directly or through identifiers linked to them (http://www.nsf.gov/bfa/dias/policy/hsfaqs.jsp#exempt)). The data were entered to an electronic database by a third party before the analyses. Of 2714 questionnaires that were handed out, 2402 were found to be complete and were ultimately used for the analyses reported here.

### Questionnaire

Students were asked whether or not they were willing to work at the community level upon graduation which was interpreted as versus other career options, your willingness to provide primary care services as a community doctor as your top choice at the time you graduate. They were also asked to provide information on factors that would likely impact their stated career choice. Questions pertaining to this set of (explanatory) variables were based on a review of the existing research [10–18] along with discussions with a group of medical students, education experts, physicians, survey researchers, and health care leaders with a knowledge of China’s medical education system. The variables and questions were subjected to a validation process, including checking for item appropriateness and comprehensiveness (face and content validity) by medical students, physicians, and experts in community health reform and services as well as in survey research methodology. They reviewed the questionnaire and provided the revision advice. Then they conducted the second-round review. This process kept going until all reviewers proved that the questionnaire have clearly addressed the questions our survey tended to ask. Modifications to the questionnaires were made following this validation process. There are 25 items eventually included into this questionnaire (Additional file 1: Table S1).

### Statistical analysis

Descriptive statistics were reported for respondents’ background characteristics. Multivariate regression analyses were conducted to examine potential factors that may impact medical students’ career choice of working as GPs in community settings after graduation. We used responses to the following question posed in the survey as a means to assess the willingness of medical student respondents to work in communities: “Are you willing to choose to work in communities upon graduation?” This question allowed for 4 responses: 1) yes, 2) yes, but only temporarily, 3) no, unless no other choice, and 4) never.

For the purposes of analysis these responses were consolidated in two ways. The first approach is to combine by the first two responses and classify them as “yes” and the last two responses as a “no”. The second approach we adopted was to allow for a three-level gradation of responses, as yes (if option 1), conditional yes (if option 2) and no (options 3 and 4).

Binomial and multinomial models were correspondingly developed. A list of plausible factors, such as place of rearing, previous exposure of community health care, were used as independent variables.

For the binomial model, we ran the logistic regression to examine the influencing factors of our interest. For the three-level outcome multinomial model, we tended to use ordinal logistic regression but it violates the proportional odds assumption. The departure from this assumption might result in an incorrect analysis and conclusion [19]. So we chose a multinomial regression model instead given its less binding assumptions. To choose a compatible test with survey sampling design, we evaluated whether the model with selected variables was significantly better than the model without these variables by using the Wald test [20] and assessed the fit of the binomial model by using the F-adjusted mean residual goodness-of-fit test [21] in addition. All the data analyses were conducted using Stata 13 software (StataCorp, College Station, TX).

## Results

### Participant characteristics

We used 2402 valid responses to analyze our predicted variable. Among these students, 19.1 % of them expressed that they would be willing to work as GPs in communities after graduation, 41.5 % of them would like to use community work as a transition, 33.7 % of them consider community work only as their backup option, and 5.7 % of them firmly expressed objection to choosing to work in communities. Response rates were also calculated for the binomial model and multinomial model, respectively, and summarized in Table 1.

**Table 1:** Response rate of survey participants on their willingness to work in communities after graduation

Response rate (%)	Binomial model	Response rate (%)	Multinomial model
Yes (Students are willing to work as GPs in communities after graduation)	60.6	Yes (Students are willing to work as GPs in communities after graduation)	19.1
No (Students are not willing to work as GPs in communities after graduation)	39.4	Conditional Yes (Students are willing to work as GPs in communities temporarily after graduation)	41.5
		No (Students are not willing to work as GPs in communities after graduation)	39.4

Table 2 presents the summary characteristics of the data with the chi-square test for the binomial model. The results show that the relationship between students’ willingness to work in communities and the most indicators is significant (*P* < 0.05) except for gender, knowledge about GP, and academic performance. We achieved the similarly significant relationship for the multinomial model except for knowledge about GP.

**Table 2:** Characteristics of survey participants, by their willingness to work in communities after graduation

	Willingness: No N (%)	Willingness: Yes N (%)	Pearsonχ^2^ (*p*-value)
Demographics			
Gender			
M (1114)	450 (47.6)	654 (45.2)	1.31 (0.25)
F (1287)	495 (52.4)	792 (54.8)	
Place of rearing			
Urban (956)	449 (48.1)	507 (37.6)	25.17 (0.00)
Rural (1326)	484 (51.9)	842 (62.4)	
Year of school			
Year 1 (566)	148 (15.6)	418 (28.9)	127.96 (0.00)
Year 2 (498)	151 (16.0)	347 (24.0)	
Year 3 (485)	202 (21.4)	283 (19.6)	
Year 4 (383)	218 (23.0)	165 (11.4)	
Year 5 (460)	227 (24.0)	233 (16.1)	
Prior contact with GP profession			
Know about GP			
No (1324)	523 (55.3)	801 (55.3)	0.00 (0.99)
Yes (1070)	423 (44.7)	647 (44.7)	
Take GM class			
No (1946)	797 (85.6)	1149 (82.2)	4.75 (0.03)
Yes (383)	134 (14.4)	249 (17.8)	
Personally held value and fit			
Income of GP			
1000– ≤ 2000 RMB (186)	88 (9.3)	98 (6.8)	11.87 (−0.01)
2000– ≤ 3000 RMB (640)	231 (24.5)	409 (28.3)	
3000– ≤ 4000 RMB (784)	295 (31.3)	489 (33.9)	
> 4000 RMB (777)	330 (35.0)	447 (31.0)	
Income of specialist			
1000– ≤ 2000 RMB (186)	46 (4.9)	102 (7.1)	14.93 (0.00)
2000– ≤ 3000 RMB (640)	211 (22.4)	328 (22.7)	
3000– ≤ 4000 RMB (784)	281 (29.8)	492 (34.1)	
> 4000 RMB (777)	406 (43.0)	520 (36.1)	
Importance of GP			
Very necessary (2001)	747 (79.6)	1254 (88.3)	34.83 (0.00)
Doesn’t matter (312)	164 (17.5)	148 (10.4)	
Not necessary at all (46)	28 (3.0)	18 (1.3)	
Social prestige			
No (1300)	430 (62.5)	870 (72.0)	18.43 (0.00)
Yes (596)	258 (37.5)	338 (28.0)	
Worth 5-year Training			
No (1080)	615 (65.6)	465 (32.8)	244.66 (0.00)
Yes (1277)	323 (34.4)	954 (67.2)	
GP profession characteristics and perceptions			
Community work condition			
Very good (18)	5 (0.5)	13 (1.0)	11.87 (−0.01)
Good (56)	16 (1.8)	40 (2.9)	
Normal (590)	183 (20.1)	407 (29.9)	
Poor (1327)	586 (64.3)	159 (11.7)	
Very poor (280)	121 (13.3)	159 (11.7)	
Working at a community health center is less stress than a big hospital			
No (536)	175 (21.9)	361 (27.7)	8.78 (0.00)
Yes (1566)	624 (78.1)	888 (73.1)	
Gloomy Career Prospect for working in a community health center			
No (444)	117 (13.6)	327 (26.9)	52.85 (0.00)
Yes (1630)	742 (86.4)	888 (73.1)	
Location of school			
Eastern China (571)	61 (6.4)	510 (35.1)	259.21 (0.00)
Central China (717)	350 (37.0)	367 (25.2)	
Western China (1114)	536 (56.6)	578 (39.7)	
Academic performance			
Very good (217)	99 (10.5)	118 (8.2)	4.2 (−0.24)
Good (942)	367 (38.8)	575 (39.8)	
Normal (1057)	417 (44.1)	640 (44.4)	
Poor (173)	63 (6.7)	110 (7.6)	

### Multivariate regression analysis

Table 3 shows the results of the binomial model and the multinomial model. Both models show significant improvement over the model only with intercept. The Wald’s χ2 is 12.79 (*p* < 0.01) for the binomial model and is 8.13 (*p* < 0.01) for the multinomial model. The F-adjusted mean residual of the binomial model is 0.72 (*p* = 0.69) which approves the goodness-of-fit of this model.

**Table 3:** Results of multivariate regression analyses of influencing factors on students’ willingness to work in communities

	Binary logistic regression Base outcome: 0 = No 1 = Yes		Multinomial logistic regression Base outcome: 0 = No Outcome 1 = Conditional yes Outcome 2 = Yes			
			Outcome 1		Outcome 2	
Explanatory variables	Parameter (Std. Error)	Odds Ratio	Parameter (Std. Error)	Odds Ratio	Parameter (Std. Error)	Odds Ratio
Intercept	2.18*** (0.48)		1.76*** (.50)		0.97 (0.65)	
Gender	0.16 (0.13)	1.18	0.20 (0.13)	1.22	0.03 (0.18)	1.03
Place of rearing	0.26* (0.13)	1.29	0.22 (0.13)	1.24	0.38* (0.18)	1.47
Year of school	−0.22*** (0.05)	.81	−0.24*** (0.05)	0.79	−0.14* (0.06)	0.87
Know about GPs	0.02 (0.13)	1.02	0.03 (0.14)	1.03	0.00 (0.18)	1.00
Take GM class	0.06 (0.17)	1.06	0.07 (0.17)	1.07	.03 (.24)	1.03
Income for GPs	0.07 (0.07)	1.08	0.04 (0.07)	1.04	0.21* (0.10)	1.23
Income for specialists	−0.10 (0.07)	0.91	−0.08 (0.08)	0.92	−0.17 (0.10)	0.85
Importance of GP: less important	−0.10 (0.20)	0.91	−0.03 (0.21)	0.97	−0.35 (0.32)	0.71
Importance of GP: not important	−0.65 (0.52)	0.52	−0.39 (0.51)	0.68	−1.87 (1.25)	0.15
Social prestige	0.00 (0.15)	1.00	-.06 (.17)	0.94	.23 (.21)	1.26
Worth 5-year training	1.40*** (0.13)	4.07	1.26*** (.14)	3.53	1.91*** (0.18)	6.74
Community work condition	−0.25* (0.11)	.78	−0.23* (0.11)	0.80	−0.31* (.14)	0.73
Less stress	0.00 (0.14)	1.00	0.07 (0.15)	1.07	−0.18 (0.19)	0.84
Gloomy career development	−0.31* (0.15)	0.73	−0.19 (0.16)	0.83	−0.62** (0.21)	0.54
School: Medical II	−1.89*** (0.35)	0.15	−1.61*** (0.36)	0.20	−2.93*** (0.52)	0.05
School: Medical III	−2.11*** (0.24)	0.12	−1.94*** (0.25)	0.14	−2.62*** (0.29)	0.07
Academic performance: good	0.50* (0.23)	1.65	0.49* (0.23)	1.64	0.50 (0.37)	1.65
Academic performance: normal	0.37 (0.23)	1.45	0.28 (0.24)	1.33	0.68 (0.37)	1.97
Academic performance: poor	0.18 (0.33)	1.20	0.00 (0.35)	1.00	0.65 (0.47)	1.92

^*^ *p* < 0.05

^**^ *p* < 0.01

^***^ *p* < 0.001

#### Binomial model

The binomial model shows that students’ willingness has a negatively significant association with several factors. For students whose year of school increases 1 year, the odds for them to be willing to work in communities decreases 19.5 % (*p* < 0.001). For each unit of decrease in the students’ evaluation of the working conditions in communities, the odds for them to be willing to work in communities decreases 21.8 % (*p* = 0.019). Students perceiving gloomy career development prospects in communities are less likely to choose to work in communities (Odds Ratio (OR) = 0.733, *p* = 0.041) than those who have a positive perception. Students from School II and III are less likely than students from School I to choose to work in communities (OR = 0.151 and OR = 0.121, respectively, both *p* < 0.001).

Some factors have positively significant impact on students’ willingness when holding other covariates constant. Students from rural areas are more likely to be willing to choose to work in communities than those from urban areas (OR = 1.294, *p* = 0.045), as are students who see a career in community health career as worth their 5-year training versus those who deem it unworthy (OR = 4.069, *p* < 0.001). And students who evaluate their academic performance as “good” are more likely than those evaluate it as “excellent” to be willing to work in communities (OR = 1.645, *p* = 0.030).

#### Multinomial model

In general, the results of the multinomial regression model were consistent with the findings of the binomial model except for the variable of perceived income for GPs where no significant result was found in the binomial model. In the multinomial model, students’ expectations for the income of GPs and their willingness to work in communities are significantly associated in a way that, when GPs’ salary increases 1 unit the odds of choosing “yes” to work in communities would increase 20.5 % (*p* = 0.040). Besides the income variable, students from a rural hometown expressed definite willingness to working in communities (Outcome 2: OR = 1.468, *p* = 0.037). Same as the binomial model, students who deem a community health career worth their 5-year training would be more likely to choose both “conditional yes” and “yes” with *p* < 0.01 (OR = 3.529 and OR = 6.740, respectively). The results for academic performance are similar to those obtained in the binomial model: respondents evaluating their performance as “good” are more likely to choose to work in communities (with “conditional yes”) than those who evaluate themselves “excellent” (OR = 1.637, *p* = 0.035).

The negative relationships are indicated in the following results. For students whose year of school increases 1 year, the odds for them to choose “conditional yes” and “yes” to work in communities decreases 21.4 % (*p* < 0.01) and 13.0 % (*p* = 0.025) respectively. And as students’ evaluation of the work conditions in communities decreases 1 unit, the odds for them to be willing to work temporarily in communities decrease 20.2 % and for them to be willing to work in communities decrease 26.6 % (*p* = 0.027). The perception regarding community career development is also significantly associated with “yes” option. Those perceiving gloomy career development prospects in community services are less likely to be willing to choose to work in communities (Outcome 2: OR = 0.537, *p* < 0.01). The school variable shows that students from School II & III are less likely than students from School I to be interested in a community health career even just temporarily (Outcome 1: OR = 0.200 for School II, OR = 0.144 for School III, *p* < 0.01; Outcome 2: OR = 0.053 for School II, OR = 0.072 for School III, *p* < 0.01).

## Discussions

This study highlighted many of the same factors at play in China as found in previous studies that focused on the career choices of North American medical students such as place of rearing [12] and the importance of prior exposure [18, 22]. Medical students willing to choose to pursue a career as GP in community health services tend to come from a rural background, have been more exposed to community health reform, and consider GP to be a valuable profession.

Unlike the findings in other studies [22, 23], marital status is not a strong factor in China given that medical education is essentially undergraduate training. Most students start medical college at the age of 18 or at an even younger age. They are not allowed to get married if they have not reached Chinese legal age for marriage (22 years for males, and 20 years for females). Marriage in college, thus tends to be very rare.

In contrast with some study that showed income may not be an influential factor [12], this study showed the important effect of income in medical students’ choice in China [17]. The level of income expectation for the GP profession has a significant impact on students’ career choice in this study. The higher the income expectation, the more likely students would be willing to choose to work in communities.

### Origin place

Students from rural areas would be more likely to choose to work in communities than those from urban areas. This phenomenon may be justified by two aspects: 1) students from rural areas have more prior exposure with basic health services so they tend to choose what they are familiar with or perceive important; 2) the level of difficulty is higher for rural students to find a job in urban areas which may make them take the work in urban community health centers in a more valuable way.

### Year in school

Students in a more senior grade are less likely to choose to work in communities. Maybe students in a lower grade do not think about and compare the career choice as much as students in a higher grade do. With the increase of years in school, students are more likely to be exposed to and affected by negative attitudes about community practice from peers and teachers, especially clinicians in hospitals. Hence, students in a senior year would be more likely to be selective in their choice and rank a community job lower than a position in a big hospital. If the curriculum or school education should have more impact on senior students’ choice due to their longer time in school, it seems to have failed in leading students toward basic health services.

### Length of training

As the majority of current community doctors only received 3 years of training, we asked these surveyed students in 5-year medical program to evaluate whether they think it is worthwhile or not for them to work in communities after 5 years of training. The responses were positively significant indicating that students consider GP a valuable profession, which suggest the possibility of bringing college graduates into community health services.

### Community perception

Students’ perception on working in communities seems to influence their career choice. This result implies that improvements in community healthcare infrastructure and certain incentives in career development opportunities can help attract more medical graduates to work in communities after graduation.

### School characteristics

The variable of school location serves as both medical school characteristic factor and location-specific community health reform factor. Students from School I were more willing to choose community health careers than students from the other two schools for several reasons, such as intensity of demand for care, job opportunities, personal interest, etc. Among all possible reasons, the exposure to local community health reform is especially implied by school variable. The emphasis on community health and its associated GP profession is a newly developed initiative in China. School I is located in a city with a leading pilot project in community health services. Therefore students there are more often exposed to community health reform and encouraged to commit to a community health career.

The school variable has another implication on students’ college entrance exam performance since three schools have different admission scores. School I has the lowest entry score. Students from School I are more likely to choose to work in communities also implies that those with poorer performance in their college entrance exam tend to choose family medicine, which conforms to the existing evidence on the inverse relationship between the Medical College Admission Test (MCAT) scores and students’ choice of family medicine or generalist careers in the US [24]. So students’ current academic performance was surveyed as well, and the results showed that students with self-rated poor academic performance are more likely to choose a career in community. The underlying reason may indicate the inferior perception of working in communities among those students. To change this stigma, the social recognition and professionalization for GP specialty need to be improved.

### Limitations

A survey study can barely circumvent the gap between the stated preference and the actual behavior of surveyed population. Answers to the survey questions are mostly subjective responses. Also the phrasing of questions may affect the accuracy of answers. We cannot examine whether those factors truly affect medical students’ choice in their actual career decision upon graduation. So a longitudinal study is recommended for future research. In addition, although we have a large sample size from three schools in representative geographic regions, in order to increase the generality of the study, a larger sample size and more schools are suggested.

## Conclusions

Career choices of medical school graduates could be impacted by changing the factors that influence their decisions: To improve the social recognition of GP profession to change the stigma in the inferior perception of community practice; To increase students’ exposure to community health reform and to improve their awareness of the importance of community practice. Improvements in community healthcare infrastructure, career development opportunities, and financial incentives can help adjust negative perceptions and attract students. Besides reforming community health careers to satisfy the desires of medical students, the alternative policy is to recruit those who are more likely to choose to work in communities in the first place. Many researches have suggested that community-oriented intervention at medical schools’ recruitment is important [25–28].

Given the recent favorable policy on promoting GPs [No. 23 (2011) of the State Council], however, whether the job attributes such as salary, free education, priority in residency training, etc. will effectively attract medical graduates to work in communities is a question to be examined in our following investigations and such experiments need to be evaluated consequently. Another policy change responding to the shortage of well-qualified GPs in China is called designated training for GPs. Students receiving free training need to sign a contract promising to work as GPs after they graduate. Following the policy on designated training for GPs, how to retrain good personnel is another concern that needs to be studied and tested in practice.

## Abbreviations

GP, General Physician; MCAT, Medical College Admission Test; OR, Odds Ratio

## Additional file

Additional file 1: Table S1. Questionnaire of medical student survey on their attitudes towards general physician profession in community health centers. (DOCX 19 kb)

## References

[1] Hung LM, Shi L, Wang H, Nie X, Meng Q (2013). Chinese primary care providers and motivating factors on performance. Fam Pract.

[2] Starfield B, Shi L, Macinko J (2005). Contribution of primary care to health systems and health. Milbank Q.

[3] Macinko J, Starfield B, Shi L (2007). Quantifying the health benefits of primary care physician supply in the United States. Int J Health Serv.

[4] Hung LM, Rane S, Tsai J, Shi L (2012). Advancing primary care to promote equitable health: implications for China. Int J Equity Health.

[5] The Ministry of Health, Department of Human Resources and Statistics Information Center (2007). 2006 China human resources for health report.

[6] Hu G, Zhang L, Sun Z (2008). The paradox of China’s health workforces: oversupply vs. scarcity. J Public Health Policy.

[7] Pepper CM, Sandefer RH, Gray MJ (2010). Recruiting and retaining physicians in very rural areas. J Rural Health.

[8] Brooks RG, Mardon R, Clawson A (2003). The rural physician workforce in Florida: a survey of US- and foreign-born primary care physicians. J Rural Health.

[9] Xu D, Sun B, Wan X, Ke Y (2010). Reformation of medical education in China. Lancet.

[10] Osborn EHS (1993). Factors influencing students’ choices of primary care or other specialties. Acad Med.

[11] Council on Graduate Medical Education (U.S.), U.S. Department of Health and Human Services, Public Health Service, Health Resources and Services Administration. Council on Graduate Medical Education: fifth report: women & medicine. Rockville, MD. 1995.

[12] Kassebaum DG, Szenas PL, Schuchert MK (1996). Determinants of the generalist career intentions of 1995 graduating medical students. Acad Med.

[13] Wade ME (2007). Influence of hometown on family physicians’ choice to practice in rural settings. Fam Med.

[14] Horner RD, Samsa GP, Ricketts TC (1993). Preliminary evidence on retention rates of primary care physicians in rural and urban areas. Med Care.

[15] Ellsbury KE, Doescher MP, Hart LG (2000). US medical schools and the rural family physician gender gap. Fam Med.

[16] Hegney D, McCarthy A, Rogers-Clark C, Gorman D (2002). Why nurses are attracted to rural and remote practice. Aust J Rural Health.

[17] Mutha S, Takayama JI, O’Neil EH (1997). Insights into medical students’ career choices based on third- and four-year students’ focus-group discussions. Acad Med.

[18] Wright B, Scott I, Woloschuk W, Brenneis F (2004). Career choice of new medical students at three Canadian Universities: family medicine versus specialty medicine. CMAJ.

[19] McCullagh P (1980). Regression models for ordinal data (with discussion). J R Stat Soc.

[20] Sribney W. Why should I not do a likelihood-ratio test after an ML estimation (e.g., logit, probit) with clustering or pweights? 2005. http://www.stata.com/support/faqs/statistics/likelihood-ratio-test/. Accessed 22 Dec 2015.

[21] Archer K, Lemeshow S (2006). Goodness-of-fit test for a logistic regression model fitted using survey sample data. Stata J.

[22] Ernst RL, Yett DE (1985). Physician location and specialty choice.

[23] Newton DA, Grayson MS, Whitley TW (1998). What predicts medical student career choice?. J Gen Intern Med.

[24] Owen JA, Hayden GF, Connors AF (2002). Can medical school admission committee members predict which applicants will choose primary care careers?. Acad Med.

[25] Martini CJM, Veloski JJ, Barzansky B, Xu G, Fields S (1994). Medical school and student characteristics that influence choosing a generalist career. JAMA.

[26] Rabinowitz HK (1988). Relationship between US medical school admission policy and graduates entering family practice. Fam Pract.

[27] Senf JH, Campos-Outcalt D, Watkins A, Bastacky S, Killian C (1997). A systematic analysis of how medical school characteristics relate to graduates’ choices of primary care specialties. Acad Med.

[28] Basco WT, Buchibinder SB, Duggan AK, Wilson MH (1998). Association between primary care-oriented practices in medical school admission and the practice intentions of matriculants. Acad Med.

