# Supplementary material for: Attitudes towards primary care career in community health centers among medical students in China
Source: BMC Fam Pract. 2016 Jul 16;17:75. doi: 10.1186/s12875-016-0472-5 (PMC4947335; doi:10.1186/s12875-016-0472-5)
Supplement: Additional file 1: Table S1. — Questionnaire of medical student survey on their attitudes towards general physician profession in community health centers. (DOCX 19 kb) [file 12875_2016_472_MOESM1_ESM.docx]

**Table 1. Questionnaire of Medical Student Survey on their Attitudes towards General Physician Profession in Community Health Centers**

**Instruction：**

1. Please choose one selection only, unless otherwise identified.

2. GP = General Physician = community doctor

**Background information:**

1. Gender: A. Male B. Female

2. Origin: A. Urban B. Rural

3. Academic year： A. 1^st^ year B. 2^nd^ year C. 3^rd^ year D.4^th^ year E. 5^th^ year

4. Academic performance: A. Excellent B. Good C. Normal D. Poor

5. Do you have any family member working in medical field? A. Yes B. No

6. Reasons to choose a medical career (**can choose more than 1**):

A. Personal interest
B. Job security
C. Prestige and social respect of medical profession
D. Job stability
E. High income
F. Not a personal choice (advice or request by family or teacher)
G. Other reason (please specify):__________________

**Your opinions on doctors’ income：**

1. What monthly income (including compensation and all other income sources) should a new medical graduate receive (in RMB)?

A. 1000-2000 B. 2000–3000 C. 3000–4000 D. >4000

2. What do you think about the income level of most physicians?

A．Too high B. Slightly high C. Fair D. Slightly low E. Too low

3. Physicians can be generally categorized as GPs and specialists. In your opinion, what should be the monthly income level of these two kinds of physicians (in RMB)?

GPs: A. 1000-2000 B. 2000–3000 C. 3000–4000 D. >4000

Specialists: A. 1000-2000 B. 2000–3000 C. 3000–4000 D. >4000

4. Your satisfaction with the current income level of GPs：

A. Very satisfied B. Satisfied C. So so D. Unsatisfied E. Very unsatisfied
F. Don’t know

**Education in general medicine：**

1. Before taking this survey, did you know about GP profession?

A. Yes B. No

2. Does your institution have curriculum in general medicine?

A. Yes B. No C. Don’t know

3. Have you ever taken a course in general medicine? (including required and elective courses)

A. Yes (____credits) B. No

4. What do you think about the necessity of GP profession?

A. Very necessary B. Doesn’t matter C. Not necessary at all

**Your opinions on working in community health centers:**

1. What’s your opinion about a graduate with five years of medical training to work at a community health center upon graduation?

A. Worthwhile B. Not worthwhile

2. What you think about the work condition in most community health centers?

A．Very good B. Good C. Normal D. Poor E. Very poor F. Don’t know

3. In your opinion, what are the advantages of working at a community health center as opposed to a big hospital? (**can choose more than 1**):

A. Good work environment B. Less stress C. Good interpersonal relationships

D. Good income E. All worse than big hospitals

4. What you think about the career development prospect for a medical graduate in a community health center?

A．Very good B. Good C. Normal D. Poor E. Very poor F. Don’t know

**Your career plan：**

1. Would you choose to be a GP if the 3-year residency is not enforced for GP?

A. Yes B. No

2. Would you choose to be a GP if the 3-year residency is enforced for GP?

A. Yes B. No

2.1. If not, please rank your other specialty choice (“1”represents your top choice）:

Internal medicine_____ Pediatric____ Surgery____

E.N.T.____ OBGYN____ Dermatology____

Other, please specify:_________________________

3. Are you willing to choose to work in communities upon graduation?

A. Yes B. Yes, but only temporarily C. No, unless no other choice D. Never

4. In your opinion, which of the following factors make graduates of five-year medical training unwilling to work at community health centers and other basic health service units? (**can choose more than 1**):

A. Poor income B. Heavy workload C. Gloomy career prospect D. Low prestige

E. Don’t know F. Other (please specify):____________________

5. What you think about the negative comments about physicians in China nowadays:

A. Very fair B. Fair C. Neutral D. Unfair E. Very unfair
F. Don’t know

6. Which of the following improvements would make you willing to choose GP profession after you graduate? (**can choose more than 1**):

A. Income not less than specialists

B. Improving work conditions and environment

C. Special compensation policies for housing, children’s education, etc.

D. Improving social respect

E. Providing training and continuing education opportunities

F. Other (please specify):______________________

**Table 2. Response Rate of Survey Participants on their Willingness to Work in Communities after Graduation**

| **Response Rate (%)** | **Binomial Model** | **Response Rate (%)** | **Multinomial Model** |
| --- | --- | --- | --- |
| **Yes** (Students are willing to work as GPs in communities after graduation) | **60.6** | **Yes** (Students are willing to work as GPs in communities after graduation) | **19.1** |
| **No** (Students are not willing to work as GPs in communities after graduation) | **39.4** | **Conditional Yes** (Students are willing to work as GPs in communities temporarily after graduation) | **41.5** |
|  |  | **No** (Students are not willing to work as GPs in communities after graduation) | **39.4** |

**Table 3.** **Characteristics of Survey Participants, by their willingness to work in communities after graduation**

|  | **Willingness: No**  **N (%)** | **Willingness: Yes**  **N (%)** | **Pearson**χ²  **(p-value)** |
| --- | --- | --- | --- |
| **Demographics** | | | |
| Gender | | | |
| M (1114) | 450 (47.6) | 654 (45.2) | 1.31  (0.25) |
| F (1287) | 495 (52.4) | 792 (54.8) |  |
| Place of Rearing | | | |
| Urban (956) | 449 (48.1) | 507 (37.6) | 25.17  (0.00) |
| Rural (1326) | 484 (51.9) | 842 (62.4) |  |
| Year of School | | | |
| Year 1 (566) | 148 (15.6) | 418 (28.9) | 127.96  (0.00) |
| Year 2 (498) | 151 (16.0) | 347 (24.0) |  |
| Year 3 (485) | 202 (21.4) | 283 (19.6) |  |
| Year 4 (383) | 218 (23.0) | 165 (11.4) |  |
| Year 5 (460) | 227 (24.0) | 233 (16.1) |  |
| **Prior Contact With GP Profession** | | | |
| Know about GP | | | |
| No (1324) | 523 (55.3) | 801 (55.3) | 0.00  (0.99) |
| Yes (1070) | 423 (44.7) | 647 (44.7) |  |
| Take GM Class | | | |
| No (1946) | 797 (85.6) | 1149 (82.2) | 4.75  (0.03) |
| Yes (383) | 134 (14.4) | 249 (17.8) |  |
| **Personally Held Value and Fit** | | | |
| Income of GP | | | |
| 1000-≤2000 RMB (186) | 88 (9.3) | 98 (6.8) | 11.87  (-0.01) |
| 2000-≤3000 RMB (640) | 231 (24.5) | 409 (28.3) |  |
| 3000-≤4000 RMB (784) | 295 (31.3) | 489 (33.9) |  |
| >4000 RMB (777) | 330 (35.0) | 447 (31.0) |  |
| Income of Specialist | | | |
| 1000-≤2000 RMB (186) | 46 (4.9) | 102 (7.1) | 14.93  (0.00) |
| 2000-≤3000 RMB (640) | 211 (22.4) | 328 (22.7) |  |
| 3000-≤4000 RMB (784) | 281 (29.8) | 492 (34.1) |  |
| >4000 RMB (777) | 406 (43.0) | 520 (36.1) |  |
| Importance of GP | | | |
| Very necessary (2001) | 747 (79.6) | 1254 (88.3) | 34.83  (0.00) |
| Doesn’t matter (312) | 164 (17.5) | 148 (10.4) |  |
| Not necessary at all (46) | 28 (3.0) | 18 (1.3) |  |
| Social Prestige | | | |
| No (1300) | 430 (62.5) | 870 (72.0) | 18.43  (0.00) |
| Yes (596) | 258 (37.5) | 338 (28.0) |  |
| Worth 5-year Training |  |  |  |
| No (1080) | 615 (65.6) | 465 (32.8) | 244.66  (0.00) |
| Yes (1277) | 323 (34.4) | 954 (67.2) |  |
| **GP Profession Characteristics and Perceptions** | | | |
| Community Work Condition | | | |
| Very Good (18) | 5 (0.5) | 13(1.0) | 11.87  (-0.01) |
| Good (56) | 16 (1.8) | 40 (2.9) |  |
| Normal (590) | 183 (20.1) | 407 (29.9) |  |
| Poor (1327) | 586 (64.3) | 159 (11.7) |  |
| Very Poor (280) | 121 (13.3) | 159 (11.7) |  |
| Working at a community health center is less stress than a big hospital | | | |
| No (536) | 175 (21.9) | 361 (27.7) | 8.78  (0.00) |
| Yes (1566) | 624 (78.1) | 888 (73.1) |  |
| Gloomy Career Prospect for working in a community health center | | | |
| No (444) | 117 (13.6) | 327 (26.9) | 52.85  (0.00) |
| Yes (1630) | 742 (86.4) | 888 (73.1) |  |
| **Location of School** | | | |
| Eastern China (571) | 61 (6.4) | 510 (35.1) | 259.21  (0.00) |
| Central China (717) | 350 (37.0) | 367 (25.2) |  |
| Western China (1114) | 536 (56.6) | 578 (39.7) |  |
| **Academic Performance** | | | |
| Very Good (217) | 99 (10.5) | 118 (8.2) | 4.2  (-0.24) |
| Good (942) | 367 (38.8) | 575 (39.8) |  |
| Normal (1057) | 417 (44.1) | 640 (44.4) |  |
| Poor (173) | 63 (6.7) | 110 (7.6) |  |

**Table 4. Results of Multivariate Regression Analyses of Influencing Factors on Students’ Willingness to Work in Communities**

|  | **Binary Logistic Regression**  Base outcome: 0=No  1 = Yes | | | **Multinomial Logistic Regression** Base outcome: 0=No  Outcome 1 = Conditional yes  Outcome 2 = Yes | | | |
| --- | --- | --- | --- | --- | --- | --- | --- |
|  |  |  |  | **Outcome 1** | | **Outcome 2** | |
| **Explanatory Variables** | | **Parameter**  **(Std. Error)** | **Odds Ratio** | **Parameter**  **(Std. Error)** | **Odds**  **Ratio** | **Parameter**  **(Std. Error)** | **Odds Ratio** |
| Intercept | **2.18*****  (0.48) | |  | **1.76*****  (.50) |  | 0.97  (0.65) |  |
| Gender | 0.16  (0.13) | | 1.18 | 0.20  (0.13) | 1.22 | 0.03  (0.18) | 1.03 |
| Place of rearing | 0.**26***  **(0**.13) | | 1.29 | 0.22  (0.13) | 1.24 | **0.38***  (0.18) | 1.47 |
| Year of school | **-0.22*****  (0.05) | | .81 | **-0.24*****  (0.05) | 0.79 | **-0.14***  (0.06) | 0.87 |
| Know about GPs | 0.02  (0.13) | | 1.02 | 0.03  (0.14) | 1.03 | 0.00  (0.18) | 1.00 |
| Take GM class | 0.06  (0.17) | | 1.06 | 0.07  (0.17) | 1.07 | .03  (.24) | 1.03 |
| Income for GPs | 0.07  (0.07) | | 1.08 | 0.04  (0.07) | 1.04 | **0.21***  (0.10) | 1.23 |
| Income for specialists | -0.10  (0.07) | | 0.91 | -0.08  (0.08) | 0.92 | -0.17  (0.10) | 0.85 |
| Importance of GP: less important | -0.10  (0.20) | | 0.91 | -0.03  (0.21) | 0.97 | -0.35  (0.32) | 0.71 |
| Importance of GP: not important | -0.65  (0.52) | | 0.52 | -0.39  (0.51) | 0.68 | -1.87  (1.25) | 0.15 |
| Social prestige | 0.00  (0.15) | | 1.00 | -.06  (.17) | 0.94 | .23  (.21) | 1.26 |
| Worth 5-year training | **1.40*****  **(0**.13) | | 4.07 | **1.26*****  (.14) | 3.53 | **1.91*****  (0.18) | 6.74 |
| Community work condition | **-0.25***  (0.11) | | .78 | **-0.23***  (0.11) | 0.80 | **-0.31***  (.14) | 0.73 |
| Less stress | 0.00  (0.14) | | 1.00 | 0.07  (0.15) | 1.07 | -0.18  (0.19) | 0.84 |
| Gloomy career development | **-0.31***  **(0**.15) | | 0.73 | -0.19  (0.16) | 0.83 | **-0.62****  (0.21) | 0.54 |
| School: Medical II | **-1.89*****  (0.35) | | 0.15 | **-1.61*****  (0.36) | 0.20 | **-2.93*****  (0.52) | 0.05 |
| School: Medical III | **-2.11*****  **(0**.24) | | 0.12 | **-1.94*****  (0.25) | 0.14 | **-2.62*****  (0.29) | 0.07 |
| Academic performance: good | **0.50***  (0.23) | | 1.65 | **0.49***  (0.23) | 1.64 | 0.50  (0.37) | 1.65 |
| Academic performance: normal | 0.37  (0.23) | | 1.45 | 0.28  (0.24) | 1.33 | 0.68  (0.37) | 1.97 |
| Academic performance: poor | 0.18  (0.33) | | 1.20 | 0.00  (0.35) | 1.00 | 0.65  (0.47) | 1.92 |

^*^ *p* < 0.05, ^**^ *p* < 0.01, ^***^ *p* < 0.001
